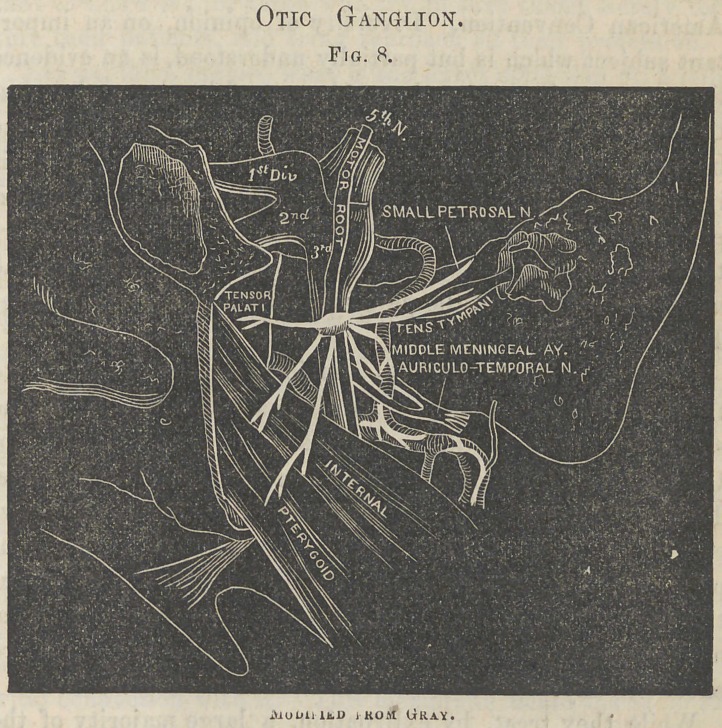# The Anatomy, Physiology, Pathology, and Remedial Treatment of the Fifth Pair of Nerves

**Published:** 1860-10

**Authors:** J. H. M’Quillen


### THE ANATOMY, PHYSIOLOGY, PATHOLOGY, AND REMEDIAL TREATMENT OF THE FIFTH PAIR OF NERVES.

BY J. H. M'QUILLEN, D. D. S.

[A synopsis presented to the American Dental Association, Washington, D. C., Aug., 1860.]

When merely regarding the dental organs as isolated portions of the human economy, the various evolutions which they undergo in the progress of their growth, the changes of maturity, the removal of the first set, and the replacement of more numerous and durable successors, and the different casualties to which these are subjected, are each and all interesting and important subjects of inquiry—but when directing attention to their sympathies, as organs of a curiously complicated structure, in alliance with the brain, through nerves which, in addition, are connected with the great sympathetic nervous system, and which brings them in close relation with the other organs concerned in nutrition, the varied influences they possess become an additional and more extended field of observation. It must be evident, therefore, to every medical and dental practitioner, that a correct knowledge of the anatomy and physiology of the fifth pair of nerves, and of the intimate relations existing between them and the nervous system generally, is indispensable to a proper appreciation of the pathological conditions to which these nerves are liable, and the sympathetic derangements frequently developed in other portions of the economy, induced primarily by dental irritation.

It may be truly said, that the first step toward the cure of disease, is to discover what the disease is, and where it is situated. The search, however, to determine what organ or function is deranged, must be most vague and indefinite without a knowledge of the structure, offices, and relations which the various parts bear to each other and the organism generally. The practice of medicine, surgery, and dentistry afford numerous examples illustrating the position, that a sound and rational practice in either department must be based upon a correct knowledge of anatomy and physiology. To no portion of the human economy, however, does this remark apply with more force than

FIG. 1.

FACIAL NERVE—From DALTON.

to the fifth pair of nerves; for, prior to the valuable discoveries of Sir Charles Bell and other investigators, with regard to the functions of the cranial nerves and the general doctrines of nervous action, it was supposed that the seat of that painful affection, *tic douloureux*, was in the *facial* nerve or portio dura of the seventh pair. Carefully conducted experiments, however, have clearly demonstrated, not only that the facial nerve is, to a very great degree, devoid of sensibility, and, therefore, can not be the seat of a painful affection, but that, emerging from the stylo-mastoid foramen, and then passing through the parotid gland, to be eventually distributed to the superficial muscles of the face, in the form of a plexus, named *pes anserinus*, it is purely a motor nerve, upon the integrity of which the expression of the countenance and the varied play of the features depend. Before this was ascertained, section of the nerve at the stylo-mastoid foramen was frequently performed for the relief of patients suffering under *tic douloureux*, but with no other result than inducing paralysis of the superficial muscles and complete loss of expression on the side of the face operated upon, the patients, after the operation, still suffering as much from the disease, and, in addition, being unable to close the eyelids, move the lips, or elevate the ala nasi of the affected side. Though essentially a motor nerve, it must be acknowledged that the branches of the portio dura possess, in addition, a certain amount of sensibility; this appears to be due, however, to the numerous anastomoses which take place at various points between it and the fifth pair of nerves.

In alluding to the importance of a knowledge of the anatomy and physiology of the fifth pair of nerves, reference has also been made to the relations existing between them and the nervous system generally. Now, to fully comprehend the cerebral, thoracic, and abdominal disturbances which unquestionably arise from dental irritation, particularly in early childhood, it is necessary that the three great divisions of the nervous system—the cerebro-spinal, the true spinal, and the great sympathetic—should be made the subject of careful study and observation. It would be out of place, and foreign to the purpose of this communication, however, to enter into a detailed consideration of these important subjects; but it may not be amiss to direct attention to them in a cursory manner, so that the relations existing between them and the fifth pair of nerves may be made apparent to all, and also that it may stimulate those who are not perfectly familiar with these subjects to cultivate a thorough and intimate acquaintance with them.

The *Cerebro-Spinal* nerves consist of *sensory* and *motor* nerves, which pass to, and proceed from, the *brain*, along its base, or *along the spinal cord*, to every part of the economy, giving sensation and voluntary motion; in other words, being the medium through which the mandates of the will are transmitted, making any part of the organism move responsive to it.

The *Reflex* or *True Spinal* nerves consist of *excitor* and *motor* nerves, which, however, only run to and proceed from the *spinal cord* and its intra-cranial continuation to the different parts of the body, and preside over the involuntary movements of the respiratory, circulatory and digestive organs. There is little or no evidence of a disposition to involuntary movements in the extremities of the human body during health or in the waking condition, as they are restrained by the controlling influence of the brain; but under the influence of certain medicines, and in certain diseased conditions of the brain and spinal cord, they become quite marked.

The *Cerebro-Spinal* and the *True Spinal Nerves* combine to form—though no *actual* union of their fibres takes place—the thirty-one pairs of nerves coming off from the sides of the spinal marrow at regular intervals, and passing out through the intervertebral foramina, to be distributed to the neck, trunk, extremities, and internal organs. These two systems are each connected with the spinal cord by two roots, anterior and posterior, the latter being the larger, and having formed upon them, in the intervertebral foramen, a ganglion. A little beyond this ganglion the anterior and posterior roots coalesce—their fibres, however, remaining distinct—and form the compound or mixed spinal nerves, which, after issuing from the intervertebral foramen, divide into anterior and posterior branches, each containing fibres from the different roots. Every spinal nerve, therefore, contains four sets of fibres, two belonging to the cerebro-spinal and two to the true spinal; the first set conveying sensation to, and volition from, the brain; the second set conveying impressions to the spinal axis, and reflex motor influence to the muscles.

FIG. 2.

SPINAL CORD, WITH NERVE ARISING BY ANTERIOR AND POSTERIOR ROOTS.—From GREY.

FIG. 3.

DIAGRAM OF SPINAL CORD AND NERVES.—From DALTON. The posterior root is seen divided at *a b*, the anterior at *c. d.*

By carefully conducted experiments, the functions of the anterior and posterior roots of the spinal nerves have been clearly demonstrated: when, for instance, the anterior root of one of the nerves is cut, loss of *motion*, but not of *sensation*, occurs in the part to which the nerve is distributed; if, on the other hand, the posterior root is cut, loss of *motion*, but not of *sensibility*, supervenes. Hence it is justly inferred that the anterior root is *motor* and the posterior root *sensory*.

The *Great Sympathetic* system consists of a double chain of

*ganglia*, commencing at the base of the brain and extending downward on each side and in front of the vertebral column to the os coccyx, where they unite at the ganglion of *Impar.*

Fig. 4.

Course and Distribution of the Great Sympathetic.
From Dalton.

This series of ganglia is the connecting medium between all the nerves of the body, and anastomoses with the cranial and spinal nerves as they emerge from their respective cavities. Thus the *ophthalmic*, the *spheno-palatine*, the *otic*, and the *submaxillary ganglia*, occupying spaces between the bones of the cranium and of the face, are connected with each other and with the three branches of the fifth pair of nerves; and each of the three *cervical*, twelve *thoracic*, five *lumbar*, and four *sacral* ganglia situated in front of the vertebral column receives motor and sensory filaments from the cerebro-spinal system. In addition to being connected with each other by slender longitudinal filaments, and with the cranial and spinal nerves, the ganglia give off branches which form numerous plexuses, and are distributed to the heart, liver, intestines, kidneys, blood-vessels, and other organs, over which the consciousness and

ANATOMY OF THE FIFTH PAIR OF NERVES.

FIG. 5.

1 IAGRAM MODIFIED AFTER TOMES.

the will have no control. The office of the sympathetic is supposed to consist, for the most part, in placing the organic in relation with the animal functions. This, however, can not be the exclusive office of the sympathetic, for, being composed of white and gray neurine, it is reasonable to look for a compound function, viz.: sensation and motion; and observation and experiment sustain the position that it does bear such relations to the viscera. After a careful investigation of all the facts connected with this system of nerves, physiologists generally have arrived at the conclusion that it exercises a threefold office: "first, that of a sensitive nerve to the parts to which it is distributed; secondly, that of a motor nerve for certain muscular parts; and thirdly, that of a nerve for the blood-vessels, supplying them with the power of contractility." In addition to this, as every ganglion is connected with other ganglia, when one of them is excited, the sensation awakened in the first may be transmitted along the entire chain, and thus arouse a train of phenomena, which may be slight or grave, according to the condition of the system and the nature of the exciting cause.

After these necessary and introductory remarks, to prevent confusion, it will be proper to take up the subject-matter of the paper in sections, commencing with a condensed, but it is trusted a comprehensive description of the *Anatomy of the Fifth Pair of Nerves.* (See preceding page.)

The Fifth, Trifacial, or Trigeminus Nerve, is analogous to the spinal nerves in its origin by two roots, anterior and posterior, or motor and sensory, the latter, also, like them, being the larger root, and having a ganglion upon it.

The *posterior root* has its origin, or physiologically speaking, its termination, in the lateral tract of the medulla oblongata, immediately behind the olivary body, and is composed of thirty or forty fasciculi, which are divisible into a hundred filaments. Tracing the root from its point of termination outward, it is found to ascend to the pons varolii, pass between its fibres, and then emerge from it in a filamentous trunk, where the pons joins the crus cerebelli; it then passes forward and expands into the large semilunar ganglion of Gasser, which rests in a depression on the upper surface of the petrous portion of the temporal bone. From the ganglion of Gasser three large trunks arise, of which the first or *ophthalmic* passes out of the skull through the sphenoidal fissure, the second or *superior maxillary* through the foramen rotundum, and the third or *inferior maxillary* through the foramen ovale.

Turning to the *anterior* or *motor root* of the fifth, it is found to consist of a very few fasciculi, which arise from the pyramidal body of the medulla oblongata, and pass through the pons varolii close to the sensory root, without, however, any union taking place between the fasciculi of the two roots. After emerging from the pons, the motor root passes under the sensory root and the ganglion of Gasser, and escapes from the skull through the foramen ovale, where it unites with the inferior maxillary branch just beyond the *otic* ganglion.

Proceeding now to trace the course of the three branches given off from the ganglion of Gasser, the first presented is

### The Ophthalmic Nerve.

The *Ophthalmic Nerve*, which comes off from the upper angle of the ganglion, is about an inch in length, somewhat flattened, and runs in the direction of the sphenoidal fissure, through which it passes to the orbit, where it divides into three branches: the frontal, lachrymal, and nasal.

The *Frontal Nerve*, the largest branch of the ophthalmic, passes forward for some distance, along the upper part of the orbit, and then divides into the supra-orbital and supra-trochlear, the first escaping from the orbit by the supra-orbital foramen, the second passing to the angle of the orbit. They are distributed to the integument of the forehead, upper eyelid, and the conjunctiva.

The *Lachrymal Nerve*, the smallest division of the ophthalmic, proceeds along the external part of the orbit to the lachrymal gland, where it divides into a superior and inferior branch, which are distributed to the upper and under surface of the gland, upper lid, and outer angle of the eye; the superior branch, in addition, anastomosing with the facial nerve.

The *Nasal Nerve* crosses the optic nerve and leaves the orbit by the anterior ethmoidal foramen, and then passes through the slit-like opening by the side of the crista galli of the ethmoid bone, and descends into the nasal cavity, where it divides into an internal and an external branch, the first to be distributed to the mucous membrane of the interior, and the second to the integument of the exterior of the nose. The nasal nerve also gives off in the orbit several branches: a *gaglionic branch*, which enters the upper angle of the *ophthalmic* ganglion; two or three long *ciliary branches*, which pierce the sclerotic coat of the eyeball, and are distributed to the iris; and an *infra-trochlear branch*, which passes to the inner angle of the eye, to be distributed to the lachrymal sac.

FIG. 6.

OPHTHALMIC GANGLION.

FIFTH PAIR OF NERVES.—From SIR CHARLES BELL. 1. small root; 2. large root, with ganglion of Gasser; 3. ophthalmic nerve; 4. superior maxillary; 5. inferior maxillary; 6. chorda tympani; 7. facial nerve; 8. ophthalmic ganglion; 9. Meckel's ganglion; 10. submaxillary ganglion; 11. submaxillary gland; 12. lingual nerve; 13. inferior dental.

The *Ophthalmic*, *Lenticular*, or *Ciliary Ganglion*, is a small, flattened, quadrangular body of a reddish gray color, (like other sympathetic ganglia,) situated within the orbit, in close proximity to the optic nerve. It unites by communicating branches or roots with the carotid plexus, the *third nerve*, or *motor-oculi*, and the nasal branch of the ophthalmic nerve, and gives off about ten short ciliary nerves from its anterior border, which pierce the sclerotic coat of the eyeball, and are distributed upon the iris.

### The Superior Maxillary Nerve.

The *Superior Maxillary*, or second division of the fifth nerve, comes off from the middle of the ganglion of Gasser, and pursues a horizontal course to the foramen rotundum, through which it passes to cross the spheno-maxillary fossa, and then enters the canal in the floor of the orbit, along which it runs to the infra-orbital foramen. The branches of the superior maxillary are the *Orbital*, *Spheno-palatine*, *Posterior*, *Middle*, and *Anterior Dental*, and *Infra-orbital.*

The *Orbital* or *Temporo-Malar Nerve* enters the orbit and divides into two branches, the *temporal* and *malar*. The *temporal* branch ascends along the outer wall of the cavity and anastomoses with the lachrymal branch of the ophthalmic, and then passes through a foramen in the malar bone and enters the temporal fossa, where it communicates with the facial and the temporal branch of the inferior maxillary, and is distributed to the integument covering the temple and side of the face. The *malar* branch runs along the outer wall of the orbit, and eventually escapes through a foramen in the malar bone, and anastomoses with the infra-orbital and facial nerves in the cheek.

The *Spheno palatine* branches are two in number, and pass to the ganglion of Meckel, in the pterygo-maxillary fossa.

The *Posterior Dental* branches arise from the superior maxillary nerve just before it enters the infra-orbital canal, and pass through small foramina in the tuberosity of the maxillary bone, and are distributed to the pulps of the upper molar teeth, lining membrane of the antrum, and gums.

Fig. 7.

Modified from Gray.

The *Middle* and *Anterior Dental* arise from the superior maxillary nerve in the infra-orbital canal, and pass through small foramina in the walls of the antrum, and are distributed to the pulps of the superior bicuspids, canines, and incisors, the lining membrane of the antrum, gums, and mucous membrane of the nares and palate. They also anastomose with the posterior dental.

The *Infra-orbital* branches, three in number, the *Palpebral*, *Nasal*, and *Labial*, are the terminating filaments of the superior maxillary nerve, which pass out of the infra-orbital foramen, and are distributed to the eyelids, nose, lips, and cheeks. They anastomose with the facial nerve.

SPHENO-PALATINE GANGLION AND ITS BRANCHES.

The SPHENO-PALATINE, or MECKEL'S GANGLION. The largest cranial ganglion is situated, as before stated, in the pterygo-maxillary fossa. The nerves connected with this ganglion are the *spheno-palatine*, which ascends to the superior maxillary nerve; the *palatine* ,(anterior, middle, and posterior,) which descend, pass through the posterior palatine foramen, and are distributed to the hard and soft palate, tonsils, and mucous membrane of the nares; the *nasal* and *naso-palatine*, branches, which enter the nasal fossa by the spheno-palatine foramen, and are distributed to the mucous membrane of that cavity—the naso-palatine passing forward and joining its fellow in the foramen incisivum, and forming the ganglion of *Cloquet;* and the *Vidian*, which passes backward from the ganglion through the Vidian foramen, and divides into the *superficial* and *deep petrous*, the latter joining the carotid plexus, while the former, continuing on, first enters the *hiatus* Fallopii, and passing through it to the aqueductus Fallopii, joins the facial nerve. Properly speaking, this is the motor root of the spheno-palatine ganglion, and arises from, rather than passes to, the facial nerve.

INFERIOR MAXILLARY NERVE.

The *Inferior Maxillary Nerve* comes from the lower and posterior part of the ganglion of Gasser, and passes out of the cranium through the foramen ovale, where a small ganglion, the *otic*, is found, and just at this point the motor root unites with the inferior maxillary nerve. Soon after this union the nerve divides into two branches, the external and the internal.

The external division sends off five branches, the *Masseteric*, *Deep Temporal* (two in number,) *Buccal* and *Pterygoid*, to the different muscles whose names they bear.

The internal and larger branch of the inferior maxillary divides into the *Auriculo Temporal*, the *Inferior Dental*, and the *Lingual* or *Gustatory*.

The *Auriculo Temporal* arises by two roots, and passes directly backward behind the articulation of the lower jaw, and then ascends between that joint and the ear, being covered by the parotid gland; on emerging from beneath the gland, it divides into the *anterior* and *posterior temporal*, which are distributed to the integuments of the temporal region. Branches are also given off which pass to the parotid gland, the pina, and meatus of the ear—and it has, in addition, branches of communication with the otic ganglion and the facial nerve.

The *Inferior Dental Nerve* passes to the posterior dental foramen of the lower jaw, and running along the canal in the middle of the bone, gives off branches to the pulps and periosteum of all the lower teeth, and also sends filaments to the gums, a portion of the nerve emerging from the anterior mental foramen, and is distributed to the mucous membrane and integument of the lower lip and chin, and anastomosing with the facial nerve. Prior to entering the foramina, a small branch, the mylo-hyoid, is given off to the muscle of that name; it also sends filaments to the digastric muscle.

The *Gustatory* or *Lingual Nerve* passes downward from its origin to the side of the root of the tongue, which it enters above the submaxillary gland, and then curves forward, and, anastomosing with the hypoglossal nerve, diverges after this into several slight fasciculi, which terminate in the papillary structure of the tongue. It sends branches to the mucous membrane of the mouth, submaxillary ganglion, tonsils, and pharynx. The Chorda Tympani, a branch of the facial nerve, which arises from it in the aqueductus Fallopii, crosses the tympanum, and escapes through t e fissura Glasseri, and, uniting with the gustatory nerve, accompanies it to the submaxillary ganglion.

### The Submaxillary Ganglion.

The *Submaxillary Ganglion* (See Fig. 10, diagram of Sir C. Bell,) is a small, round, or triangular body, which lies upon the gland of the same name, close to the *gustatory nerve*, from which it derives its sensitive filaments, the motor filaments coming from the facial nerve by the chorda tympani; it also communicates by longitudinal filaments with the superior cervical ganglion, and sends branches to the submaxillary and sublingual glands and the sides of the tongue.

### Otic Ganglion.

Fig. 8.

Modified from Gray.

The *Otic Ganglion*, as already stated, is a small oval ganglion connected with the inferior maxillary nerve, near the foramen ovale. It receives filaments of communication from the carotid plexus, a sensory root from the inferior maxillary branch of the fifth pair, a motor root from the facial nerve, and sends filaments to the tensor tympani muscle aud the mucous membrane of the tympanum and Eustachian tube.

(To be continued.)

## Figures and Tables

**Fig.1. f1:**
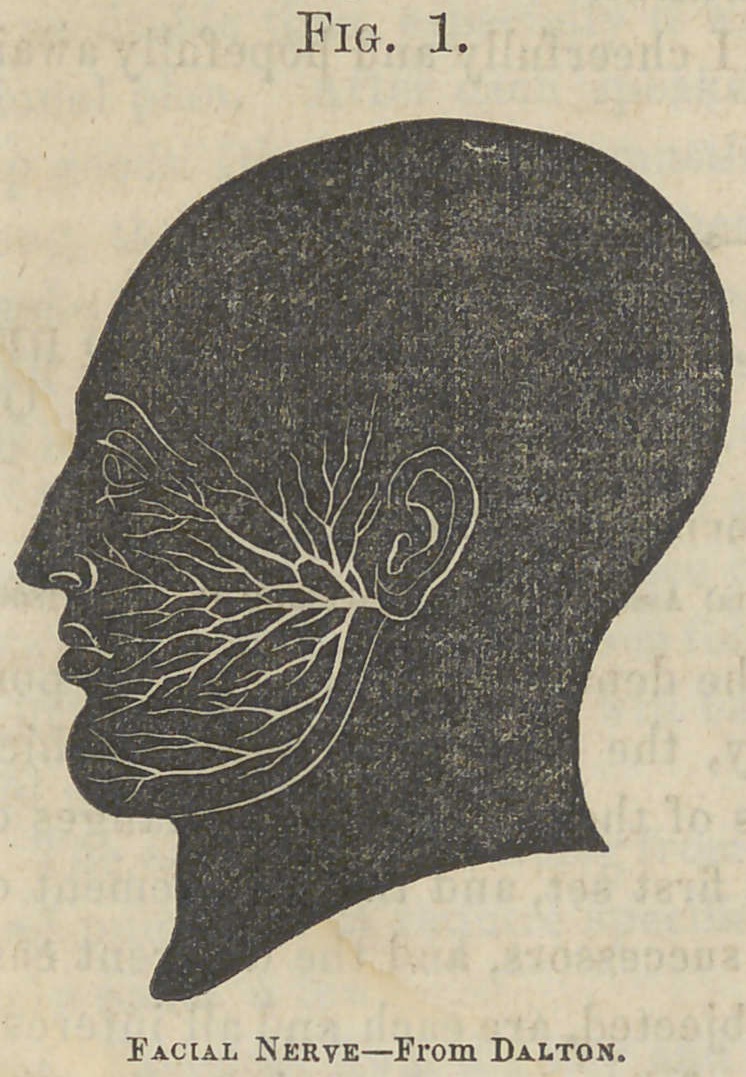


**Fig.2. f2:**
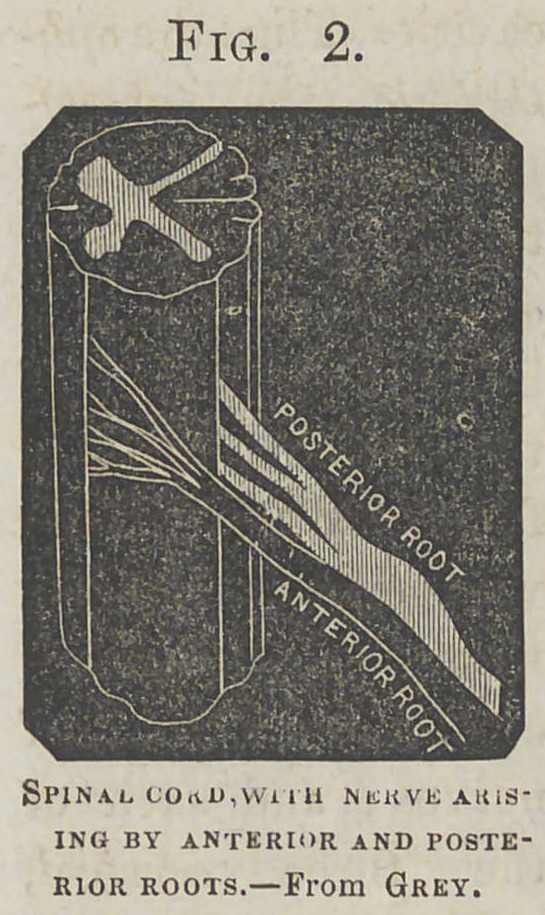


**Fig.3. f3:**
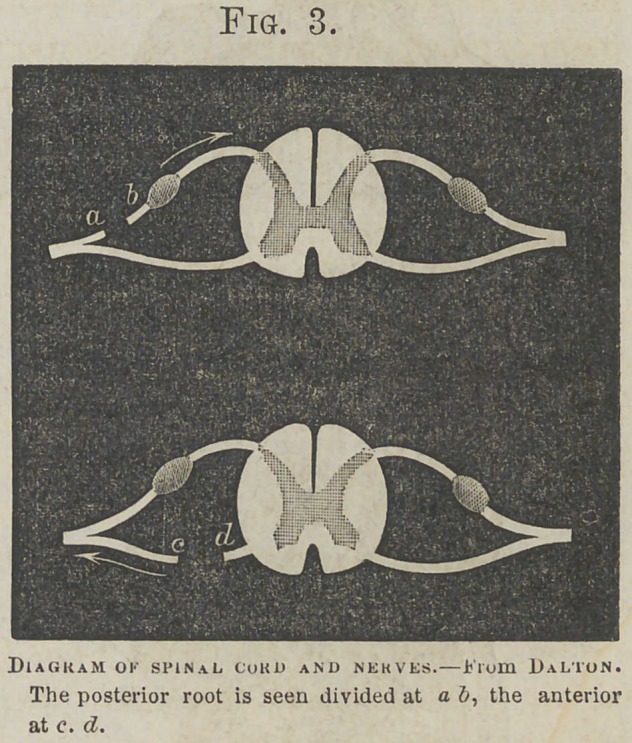


**Fig.4. f4:**
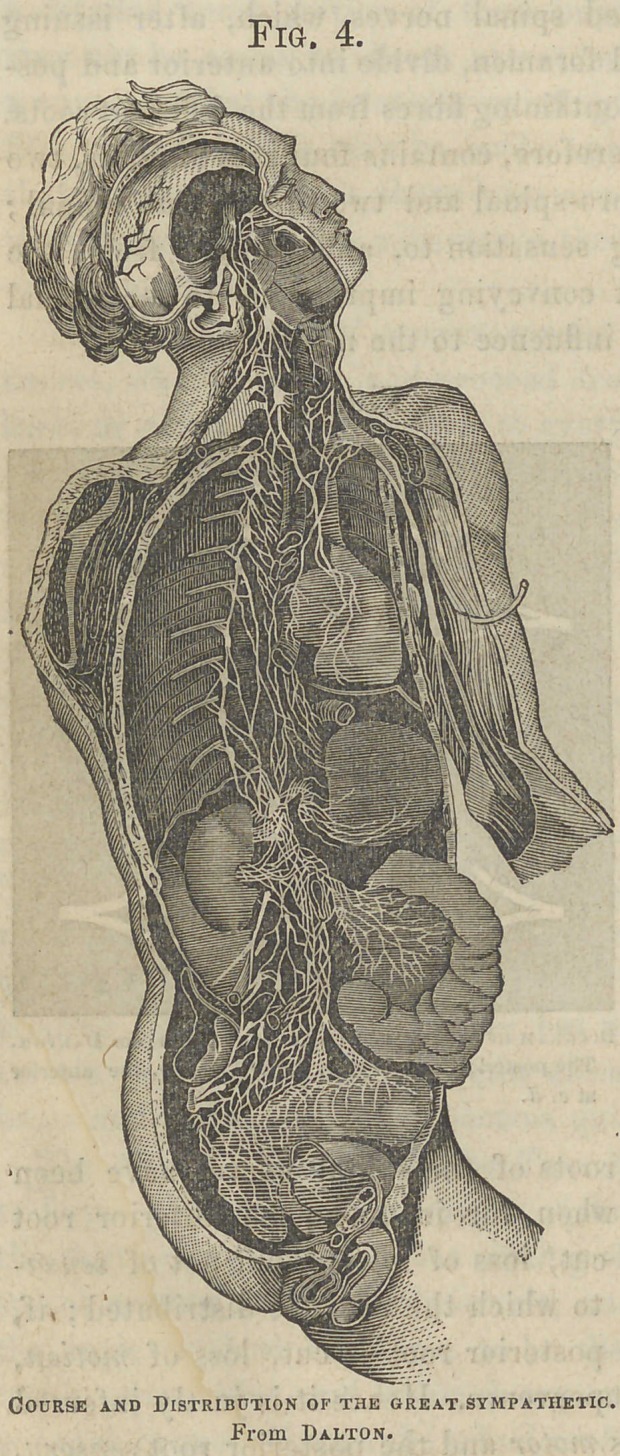


**Fig.5. f5:**
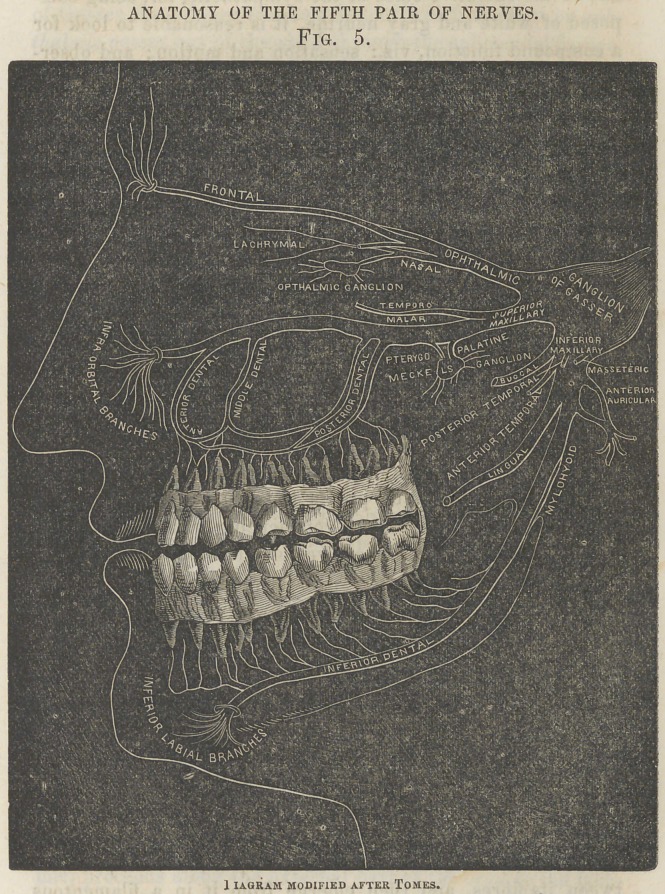


**Fig.6. f6:**
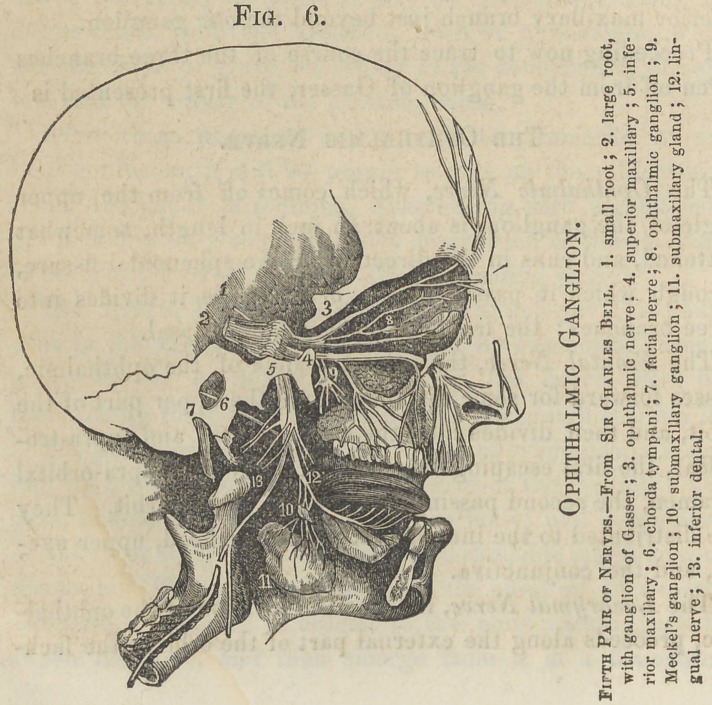


**Fig.7. f7:**
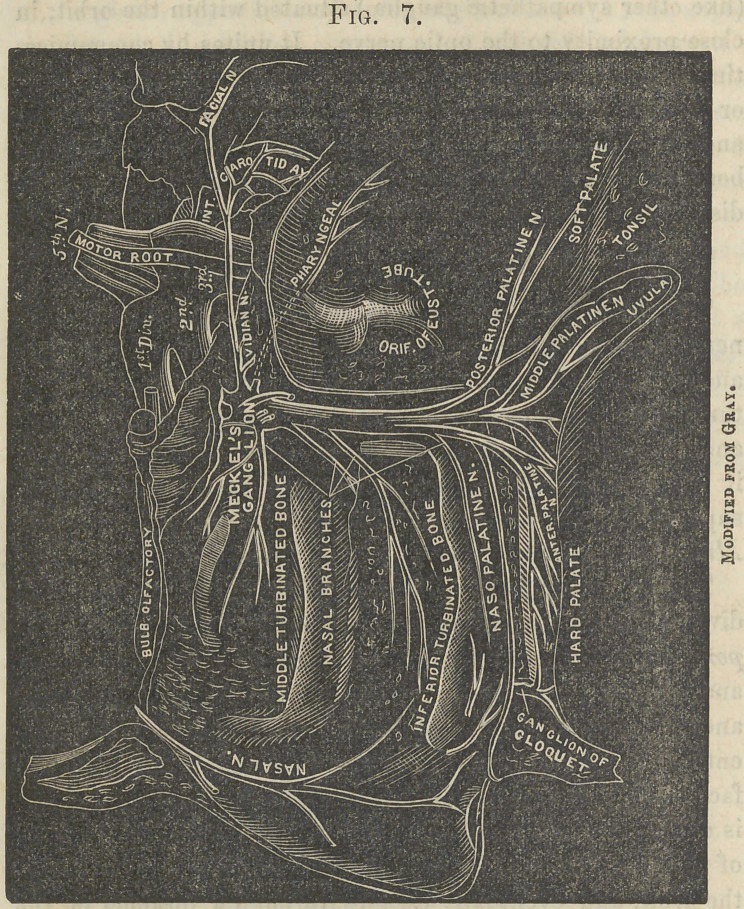


**Fig.8. f8:**